# Identification and validation of a PD-L1-related signature from mass spectrometry in gastric cancer

**DOI:** 10.1007/s00432-022-04529-6

**Published:** 2023-01-02

**Authors:** Xiancong Chen, Deli Mao, Dongsheng Li, Wenchao Li, Hongfa Wei, Cuncan Deng, Hengxing Chen, Changhua Zhang

**Affiliations:** 1 Digestive Diseases Center, The Seventh Affiliated Hospital of Sun Yat-Sen University, Shenzhen, China; 2 Guangdong Provincial Key Laboratory of Digestive Cancer Research, The Seventh Affiliated Hospital of Sun Yat-Sen University, Shenzhen, China

**Keywords:** PD-L1, Immunotherapy, Tumor microenvironment, Mass spectrometry, Machine learning

## Abstract

**Background:**

According to the guidelines, PD-L1 expression is a critical indicator for guiding immunotherapy application. According to certain studies, regardless of PD-L1 expression, immunotherapy could be advantageous for individuals with gastric cancer. Therefore, new scoring systems or biomarkers are required to enhance treatment strategies.

**Methods:**

Mass spectrometry and machine learning were used to search for strongly related PD-L1 genes, and the NMF approach was then used to separate gastric cancer patients into two categories. Differentially expressed genes (DEGs) between the two subtypes identified in this investigation were utilized to develop the UBscore predictive model, which was verified by the Gene Expression Omnibus (GEO) database. Coimmunoprecipitation, protein expression, and natural killing (NK) cell coculture experiments were conducted to validate the findings.

**Results:**

A total of 123 proteins were identified as PD-L1 interactors that are substantially enriched in the proteasome complex at the mRNA level. Using random forest, 30 UPS genes were discovered in the GSE66229 cohort, and ANAPC7 was experimentally verified as one of 123 PD-L1 interactors. Depending on the expression of PD-L1 and ANAPC7, patients were separated into two subgroups with vastly distinct immune infiltration. Low UBscore was related to increased tumor mutation burden (TMB) and microsatellite instability-high (MSI-H). In addition, chemotherapy medications were more effective in individuals with a low UBscore. Finally, we discovered that ANAPC7 might lead to the incidence of immunological escape when cocultured with NK-92 cells.

**Conclusion:**

According to our analysis of the PD-L1-related signature in GC, the UBscore played a crucial role in prognosis and had a strong relationship with TMB, MSI, and chemotherapeutic drug sensitivity. This research lays the groundwork for improving GC patient prognosis and treatment response.

**Supplementary Information:**

The online version contains supplementary material available at 10.1007/s00432-022-04529-6.

## Introduction

The ubiquitin‒proteasome system (UPS) is a significant posttranslational modification that degrades proteins under normal and pathological situations (Pohl and Dikic 2019). It is widely believed that the UPS comprises several crucial elements, including ubiquitin, ubiquitin-activating enzymes, ubiquitin-conjugating enzymes, ubiquitin-protein enzymes, deubiquitinating enzymes, and the 26S proteasome (Puvar et al. 2019). UPS mediates several biological processes, including the cell cycle, apoptosis, and the reaction to DNA damage (Wu et al. 2020). Dysregulation of this system results in the onset or development of diseases (Sharma et al. 2021), including cancer, metabolic syndrome, neurological illnesses, inflammatory disorders, and muscular dystrophy (Favaro et al. 2019; Hooijman et al. 2015; Ndoja et al. 2020). Multiple immunological components of the tumor microenvironment have been found to control carcinogenesis, making them interesting immunotherapeutic targets (Sanmamed and Chen 2018). The UPS is essential for immune activation by activating the NF-B pathway and secreting interferon gamma (Hiscott et al. 2003; Owens et al. 2020). In addition, the UPS is linked to the invasion of immune cells and tumor immune evasion (Zhu et al. 2020). Studies have shown that proteasomes contribute to the development of cytotoxic T-cell responses (Osterloh et al. 2006; Yang et al. 2020).

As a transmembrane protein, PD-L1 is to blame for the immunosuppressive tumor microenvironment (Giatromanolaki et al. 2019). Emerging research shows that diverse agents targeting ubiquitin-protein enzymes might affect PD-L1 proteasomal degradation, such as STUB1 (Mezzadra et al. 2017), while compounds targeting deubiquitinating enzymes regulate PD-L1 deubiquitination, such as CSN5 (Lim et al. 2016). UPS ultimately results in the modulation of the PD-1/PD-L1 pathway and regulates immunosuppression and antitumor effects (Hu et al. 2021).

As the fifth most prevalent cancer overall and the fourth major cause of cancer-related death, gastric cancer has risen to prominence in the recent years (Sung et al. 2021). Approximately 40–63% of patients diagnosed with gastric cancer have an elevated level of PD-L1 expression (Liao et al. 2022). Increased PD-L1 expression is related to lymph-node metastases, advanced disease, and poor prognosis in gastric cancer. In clinical studies, immune checkpoint inhibitors showed potential antitumor effects against gastric cancer. KEYNOTE-059 demonstrated that patients with PD-L1-positive gastric cancer (CPS >  = 1) had a longer median duration of response and a higher objective response rate, which led to the FDA authorizing pembrolizumab as a third-line therapy for patients with PD-L1-positive (CPS >  = 1) gastric cancer (Fuchs et al. 2018). PD-L1 expression is now regarded as a prerequisite for the clinical use of some immunotherapies (e.g., pembrolizumab) in treating gastric cancer. However, nivolumab is currently approved in Japan for treating advanced gastric cancer resistant to conventional chemotherapy, independent of the presence of PD-L1 (Joshi and Badgwell 2021). Therefore, we hypothesized that PD-L1 and other variables simultaneously affected the immunotherapy effect, and proteins interacting with PD-L1 were identified by mass spectrometry. We identified ANAPC7 concerning the PD-L1 interaction and created a full PD-L1-related signature for GC prognostic prediction. Our findings provide fresh light based on immunotherapy and tailored treatment options.

## Materials and methods

### Cell lines

The cell lines SGC-7901, BGC-803, HGC-27, AGS, SNU-719, and HEK293, as well as NK-92, were acquired from the Cell Bank of the Chinese Academy of Sciences in Shanghai, China. The cells were grown in medium with 10% fetal bovine serum (FBS), which was maintained at 37 °C with 5% CO2.

### Mass spectrometry

SFB-tagged PD-L1 and SFB-vector were transiently transfected into HEK293 cells, respectively. The cells were lysed for 30 min. The supernatant was collected by centrifugation at 12,000 g to remove debris, and it was then incubated for 12 h at 4 °C with Streptavidin Magnetic Beadsstreptavidin magnetic beads (NEW ENGLAND BioLabs, S1420S). Liquid chromatography–tandem mass spectrometry (LC‒MS/MS) was used to examine peptides, and probable PD-L1-binding motifs were found.

### GC dataset source and preprocessing

Gene expression profiles and clinical information were retrieved from the TCGA and GEO databases. The TCGA–STAD cohort includes 375 GC samples and 32 normal tissue samples, while the GSE84437 cohort contains 433 GC samples, and the GSE66229 cohort contains 300 GC samples and 100 normal tissue samples. TCGA-STAD and GSE84437 were combined to create a training cohort, whereas GSE66229 served as a validation cohort. The TCGA–STAD cohort's fragments per kilobase million (FPKM) data were transformed to transcripts per million (TPM). The "sva" R software package was used to address the batch impact. Patients with insufficient survival data were excluded.

### Random forest analyses and artificial neural network

Random forest (RF) analyses were conducted on 135 ubiquitin‒proteasome system (UPS) genes in the GSE66229 cohort, and 30 genes were chosen based on the cross-validation error threshold with the smallest value. The ntree was 500, whereas the setting seed was 123. The R software “neuralnet” and “NeuraNetTools” packages were used to construct an artificial neural network consisting of 30 genes screened by random forest using the seed 12,345,678. In addition, we created a receiver-operating characteristic (ROC) curve for the diagnostic model using the “pROC” R package.

### Molecular subtype identification

PD-L1 and ANAPC7 expression profiles were derived from the TCGA and GSE84437 merged cohort. GC samples were grouped with the use of the nonnegative matrix factorization (NMF) clustering method, with the standard “brunet” option selected and 50 iterations carried out. The value of k, the number of clusters, was set in the range of 2 to 10. The appropriate number of clusters was ultimately identified using indices such as cophenetic, dispersion, and silhouette.

### Generation of the UBscore

The possibility of overfitting was minimized with the use of the least absolute shrinkage and selection operator (LASSO) analysis implemented in the “glmnet” R package. Using a multivariate Cox analysis, we identified candidate genes for use in building a predictive model (UBscore) in the training cohort. The UBscore was determined as follows:

$$\mathrm{UBscore}=\sum \left( Coe{f}_{i}*{Exp}_{i}\right);$$

*i* represents genes selected by LASSO-COX.

### Construction and validation of a nomogram

Combining the UBscore level with common clinical indicators led to the creation of a predictive nomogram. To further evaluate the model's accuracy, calibration curves were created for 1-, 3-, and 5-year OS. The predictive capacity of the UBscore alone was compared to that of the nomogram model using a receiver-operating characteristic (ROC) curve.

### Drug susceptibility analysis

Drug susceptibility in the two risk groups was analyzed using the “pRRophetic” R package. Half-maximal inhibitory concentration (IC50) values were obtained for commonly used chemotherapeutic medications to examine the relevance of the UBscore in clinical medication selection.

### Investigation of the model’s relevance to immune checkpoint blockade

The immunophenoscore (IPS) is a term including the four major components (effector cells, MHC molecules, immunomodulators, and immunosuppressive cells) that contribute to immunogenicity and is determined using machine learning approaches without bias. The immunophenoscore file for immune checkpoint inhibitors (ICI) was obtained from The Cancer Immunome Atlas (TCIA).

### Western blotting and coimmunoprecipitation

After being washed in PBS, the cells were lysed by being combined with lysis buffer for 30 min and then centrifuged at 4 °C Celsius for 10 min at 12,000 g. SDS‒PAGE was used to separate the cleared lysates. For Western blots, samples were transferred to PVDF membranes and identified using ECL detection reagents. For coimmunoprecipitation (CoIP), IP lysis buffer was used to lyse cells (Thermo Fisher Scientific, 87,788). First, the supernatants were incubated at 4 °C overnight with anti-Flag (Abmart, M20008) and anti-PD-L1 (ABMART, M033179) antibodies, followed by a 4 h incubation at 4 °C with Protein A/G Magnetic Beads (Bimake, B23202). The beads were rinsed five times with IP lysis buffer. Then, SDS-PAGE was used to separate the samples.

### Cytotoxicity determination

Using a lactate dehydrogenase (LDH) cytotoxicity detection kit (C0017, Beyotime), the cytotoxic activity of NK-92 cells was evaluated on AGS (Vector) and AGS (ANAPC7-OE) tumor cells for 4 h. The specific death rate (%) was determined by dividing the coculture-cell well's OD values by the maximum-cell-LDH well's OD values.

### Statistical analysis

All statistical analyses were conducted using R (R 4.1.3) software. Using an unpaired, two-tailed Student’s t test, the difference between the two independent groups was determined. *P* < 0.05 was considered statistically significant.

## Result

### Detection of proteins binding to PD-L1

**Fig. 1:**
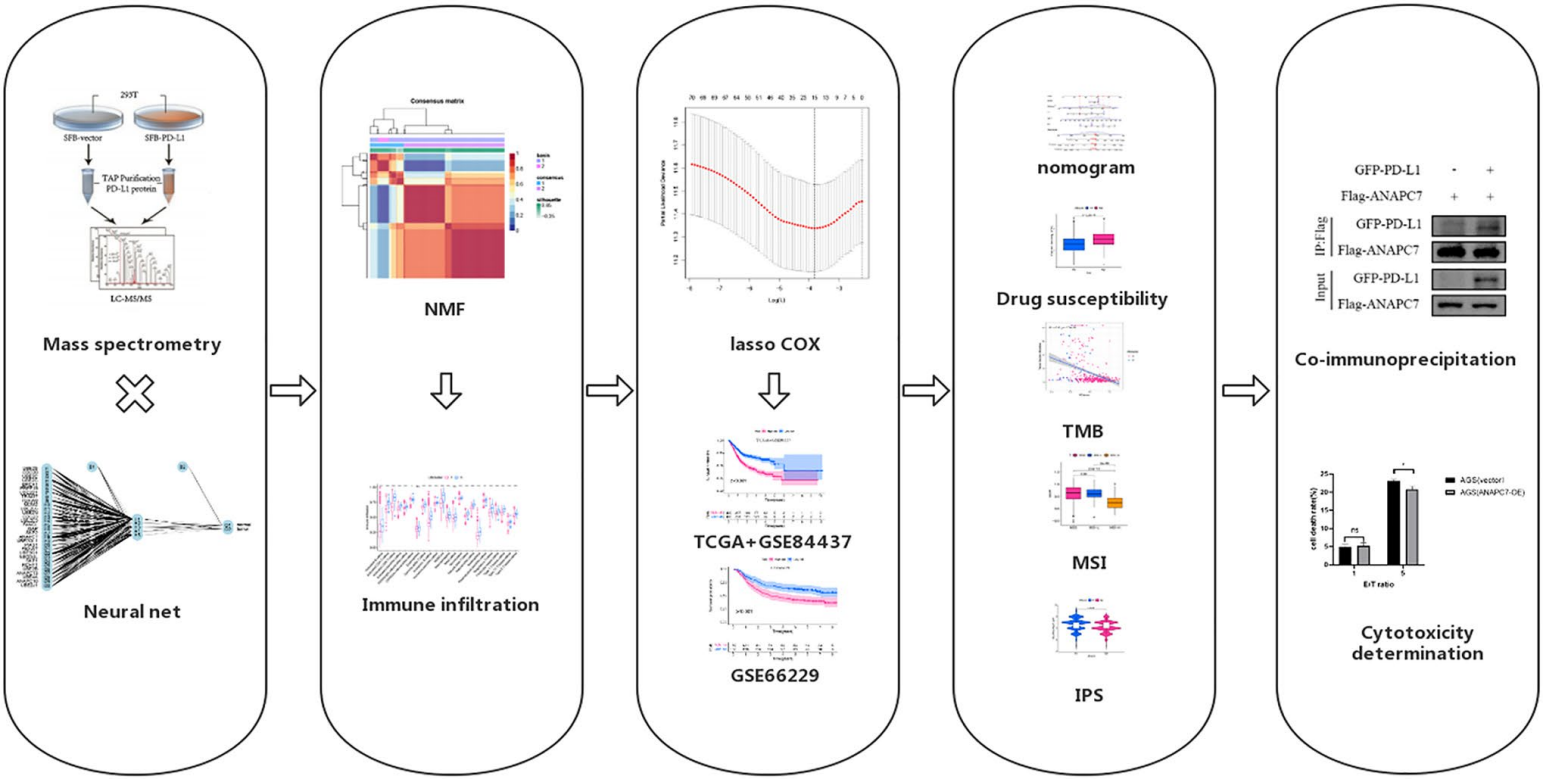
Overview of this research

**Fig. 2:**
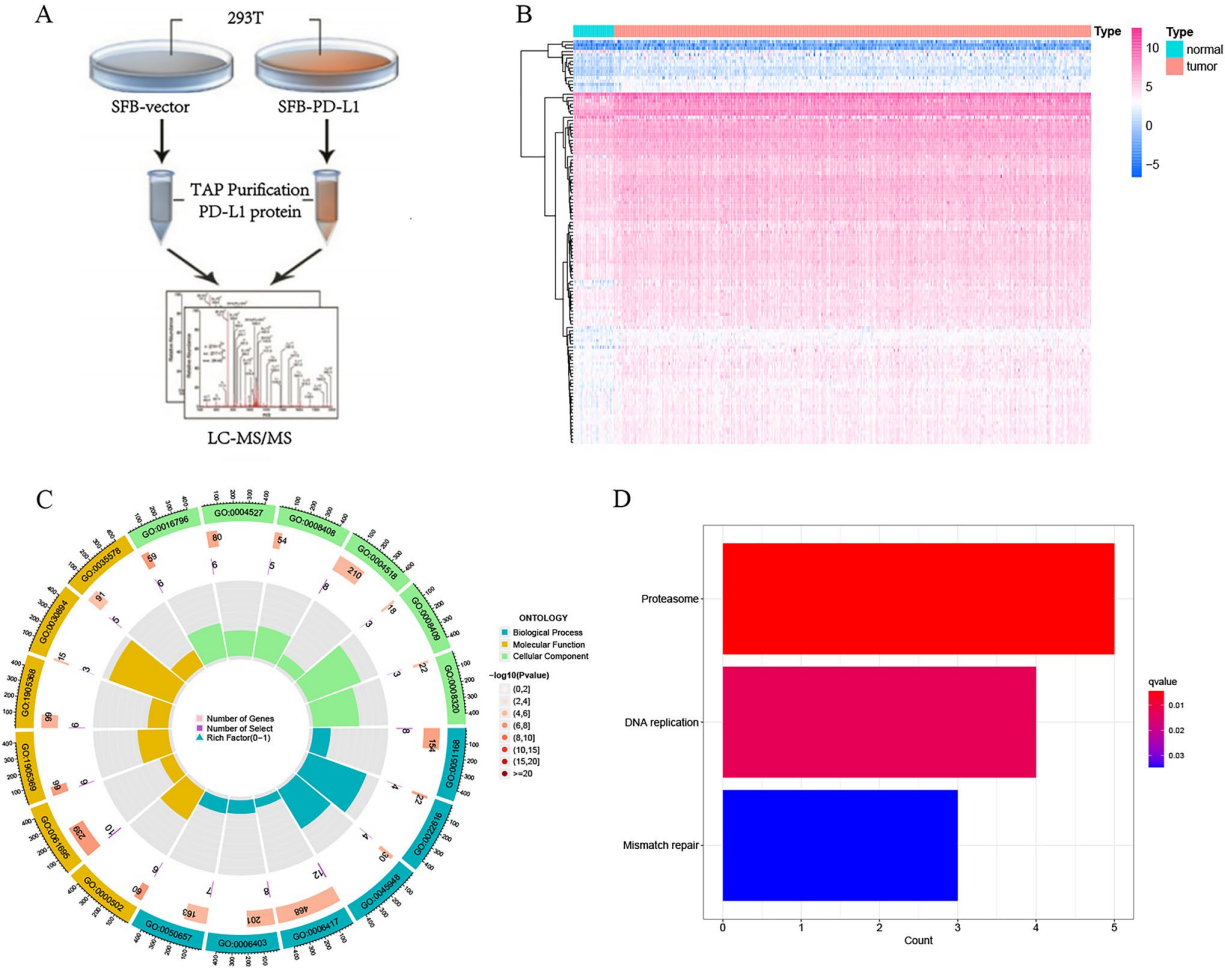
Characterization of proteins interacting with PD-L1 at the mRNA level. **A** Workflow schematic for proteomics. HEK293 cells were transfected with SFB-vector and SFB-PDL1, and the protein differences between the two groups were determined using mass spectrometry. **B** Observation of different proteins at the mRNA level in normal and malignant stomach tissues (In the TCGA database, 123 differentially expressed genes were filtered by | logFC |> 0.6, *P* < 0.05.) **C**. GO analysis of 123 DEGs. **D**. KEGG analysis of 123 DEGs

Figure 1 is a description of the study’s flowchart. First, vector and PD-L1 plasmids containing flag-tag were constructed and transiently transfected into HEK293 cells, respectively.. A total of 195 distinct proteins were examined in the mass spectrometry results (Fig. 2A and TABLE S1). The mRNA expression of these differential proteins was obtained from the TCGA cohort, and 123 differentially expressed genes (DEGs) were evaluated against the standard of | logFC |> 0.6 and *P* < 0.05; 2 genes were downregulated in tumors, and 121 genes were upregulated in cancers (Fig. 2B). To investigate functional enrichment in PDL1-related DEGs, GO molecular function enrichment analysis and KEGG pathway enrichment analysis were conducted. They were enriched in proteasome complex, protein transmembrane transporter activity, and regulation of translation, according to GO analysis (Fig. 2C). According to the KEGG enrichment study, it was mostly enriched for proteasome, DNA replication, and mismatch repair (Fig. 2D).

### Creating an artificial neural network with 30 UPS genes

**Fig. 3:**
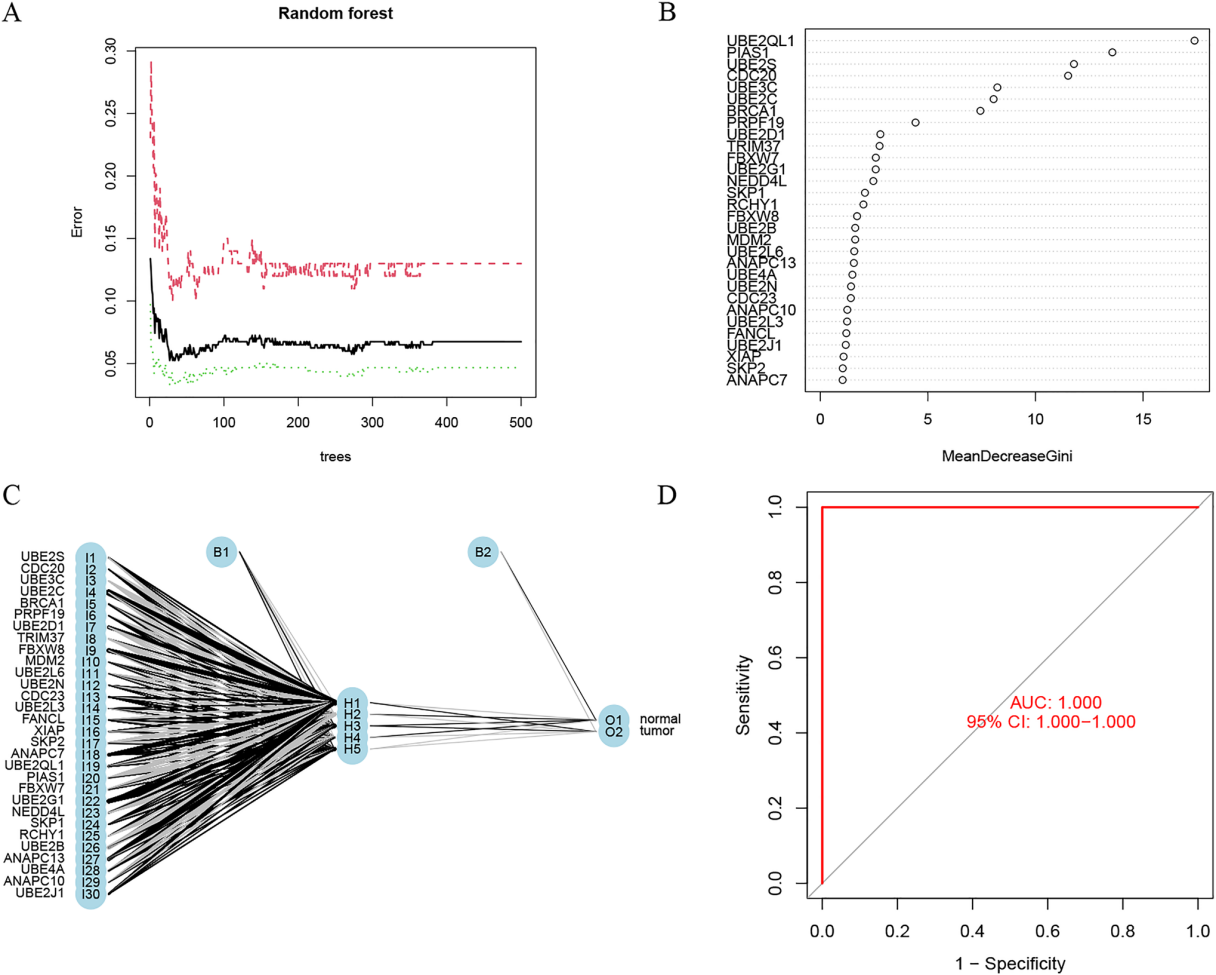
Developing an ANN model. **A** The effect of the number of decision trees on the mistake rate. The x-axis represents the decision tree, while the y-axis represents the mistake rate. **B** The Gini index in random forest modeling for the GSE66229 cohort. On a scatterplot, genetic factors are displayed along the y-axis, and significance indices are displayed along the x-axis. **C** RF-selected 30 genes were used to develop an artificial neural network. **D** ROC curves of the GC-based GSE66229 cohort diagnostic model

The ubiquitin-mediated proteolysis pathway in the KEGG provided 135 genes for the ubiquitin‒proteasome system (UPS). Random forest was used to identify the most important UPS genes in the GSE66229 cohort. The average mistake rate was determined by applying a recurrent RF classification to each conceivable number of variables. The error rate was quite low when the number of decision trees was approximately 30 (Fig. 3A), and the 30 most important genes were selected (Fig. 3B). Then, we used artificial neural networks to evaluate the diagnostic value of the 30 UPS genes (Fig. 3C). Surprisingly, the area under the curve (AUC) for the receiver-operating characteristic (ROC) curve was 1 (Fig. 3D).

### Ubiquitin pattern mediated in GC by PD-L1 and ANAPC7

**Fig. 4:**
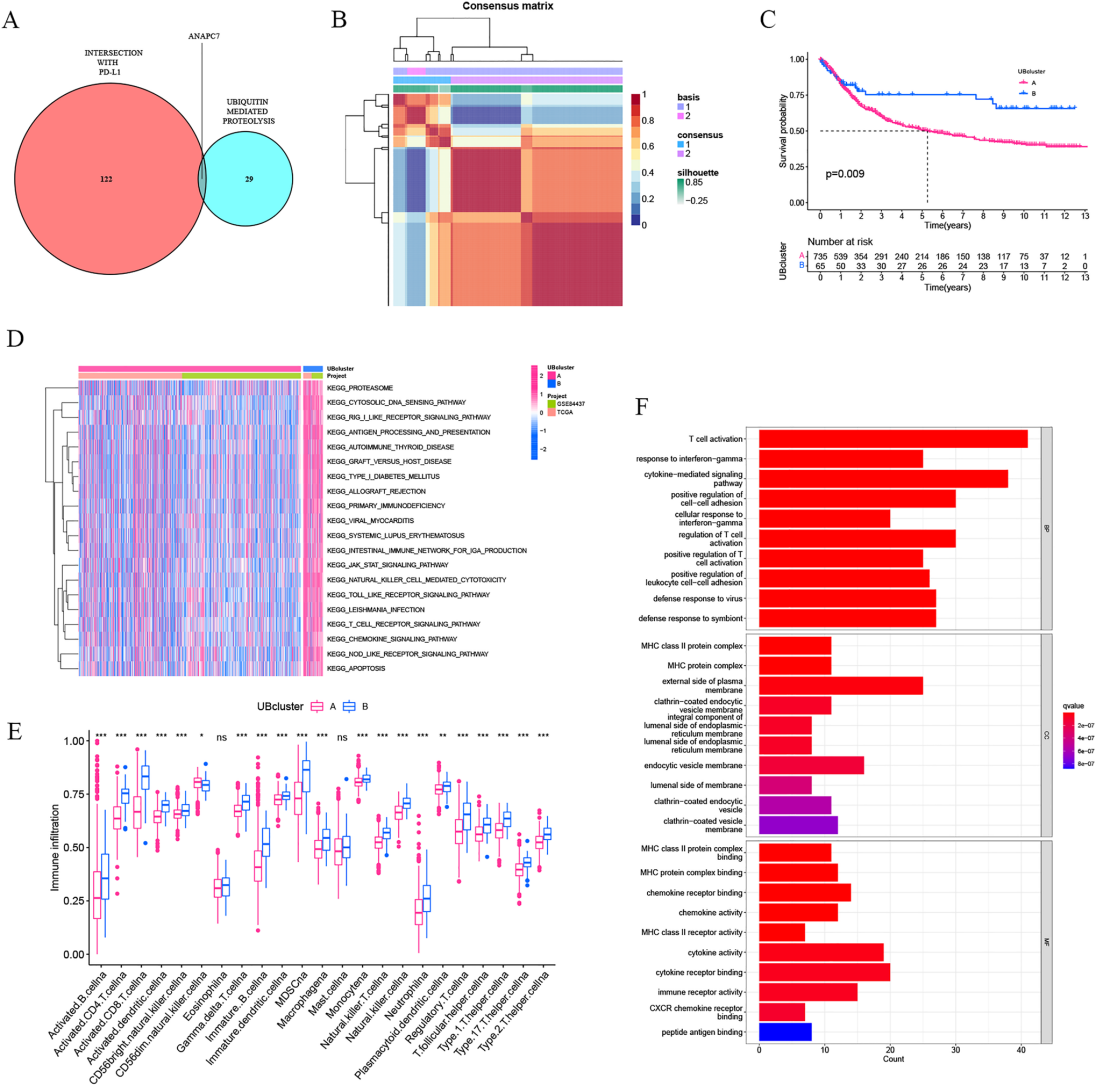
Construction and characterization of the UBcluster. **A** Gene crossover for mass spectrometry and random forest screening. **B** A heatmap of the NMF matrix identifying two clusters (k = 2) and their correlation region. **C** The overall survival of each pattern in the TCGA and GSE84437 merged cohort depicted by the Kaplan‒Meier curve. **D** GSVA demonstrated various patterns of active pathways. A route that was activated is shown in red, while a pathway that was inhibited is displayed in blue. **E** The abundance of each TME-infiltrating cell in two UBclusters. *P < 0.05; **P < 0.01; ***P < 0.001. **F** GO enrichment analyses of DEGs among the two UBclusters

ANAPC7, an E3 ubiquitin ligase, was the intersection of PDL1-related DEGs and 30 genes selected by random forest (Fig. 4A). The NMF algorithm grouped GC samples based on the expression of PD-L1 and ANAPC7 in the TCGA and GSE84437 combined cohorts. Cophenetic, dispersion, and silhouette measurements all show that the ideal number of clusters is *k* = 2 (Fig. 4B). UB cluster B had a better prognosis than UB cluster A based on overall survival (OS) (Fig. 4C).

### Immunity differences between the two ubiquitin patterns

The GSVA enrichment analysis revealed that UB cluster B was strongly enriched in molecular function-related pathways, such as proteasome, apoptosis, cytosolic DNA sensing pathway, antigen processing, and presentation. At the same time, UB cluster B revealed enriched pathways linked with immune activation, including the JAK/STAT signaling pathway, natural killer cell-mediated cytotoxicity, Toll-like receptor signaling pathway, and T-cell receptor signaling pathway (Fig. 4D). The immunologically infiltrating cells in UBcluster A and UBcluster B were then studied. As shown in Fig. 4E, immune infiltrating cells were more abundant in UBcluster B than in UBcluster A, indicating a distinction between the two cluster subtypes regarding immune cell infiltration. To further study the potential biological role of each cluster, 260 UBcluster-related DEGs with the standard of | logFC |> 1 and *P* < 0.05 were identified in the TCGA-STAD and GSE84437 cohorts using the limma program. These genes were subjected to GO enrichment studies, which revealed an enrichment of biological processes strongly associated with immunity, including T-cell activation and the MHC class II protein complex (Fig. 4F).

### Construction and verification of a risk model associated with prognosis

**Fig. 5:**
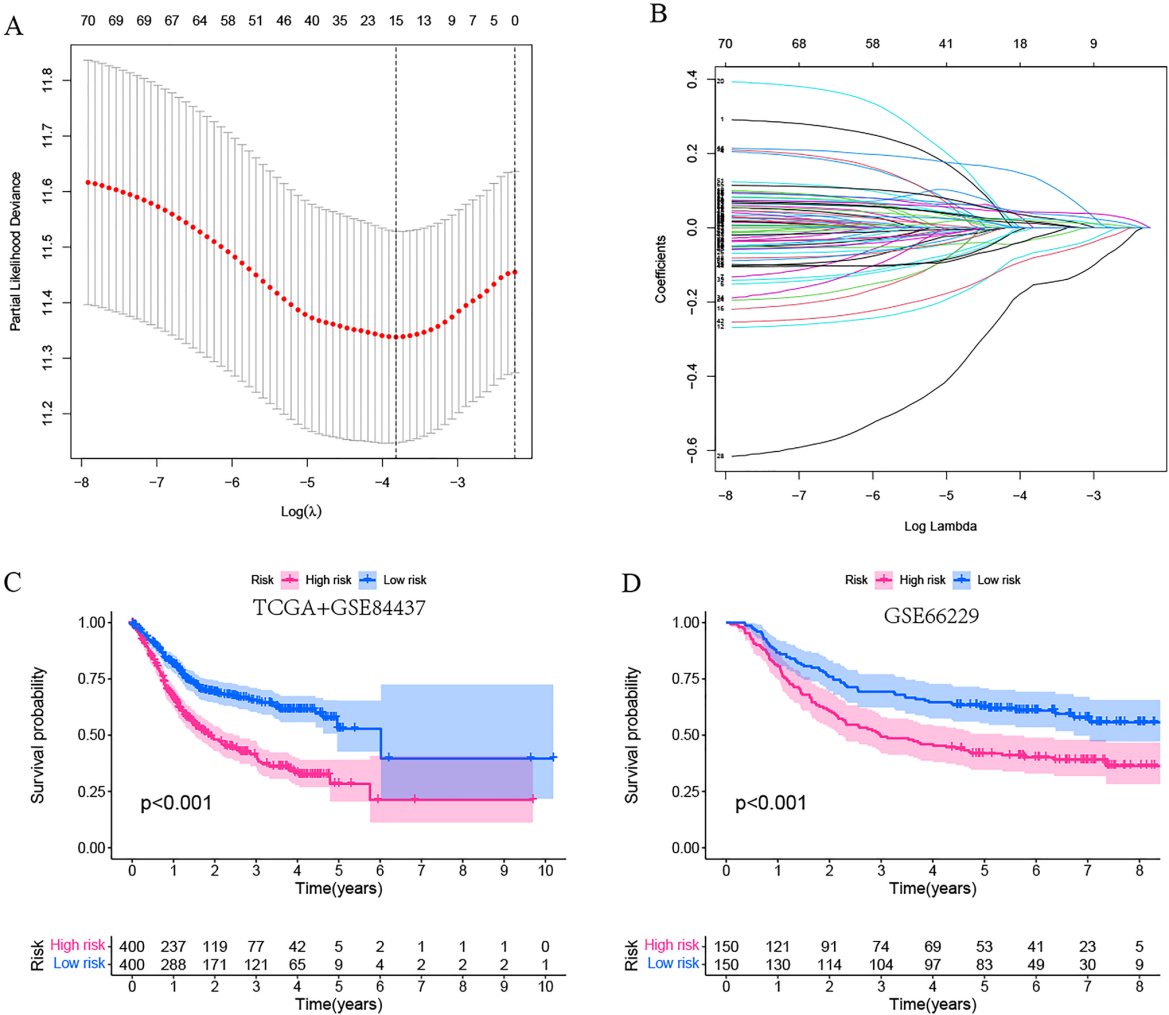
Construction and verification of the prognostic risk signature. **A**, **B** Identifying representative potential prognostic genes. **C**, **D** The Kaplan‒Meier curve was performed to assess the overall survival of GC patients in low-to-high-risk categories

Using the survival program to perform univariate Cox regression analysis on 260 UBcluster-related DEGs with *P* < 0.05, 77 genes were identified as being associated with prognosis. To further reduce irrelevant factors and produce fewer significant variables, we utilized the “glmnet” package to conduct LASSO regression to identify 15 essential genes (Fig. 5A, B). This was followed by a multivariate Cox regression analysis, which yielded 13 genes for the construction of the UBscore prognostic model. The formula for calculating the risk score (UBscore) is

$$\mathrm{Risk score}\hspace{0.17em}=\hspace{0.17em}\sum_{i=1}^{13}Coe{f}_{i}*{x}_{i};$$

*i* = {1, 2, 3,…13}. Coefi refers to the coefficients of genes in the prognostic model, whereas *x*_*i*_ represents the expression of genes. Depending on the median UBscore, patients were divided into high- and low-risk groups. In the training cohort (TCGA and GSE84437 combined cohorts) and validation cohort (GSE66229), the Kaplan‒Meier curve indicated that the overall survival of GC patients in the high-risk (high-UBscore) group was considerably lower than that in the low-risk (low-UBscore) group (Fig. 5C, D).

### Development and validation of a survival prediction nomogram

**Fig. 6:**
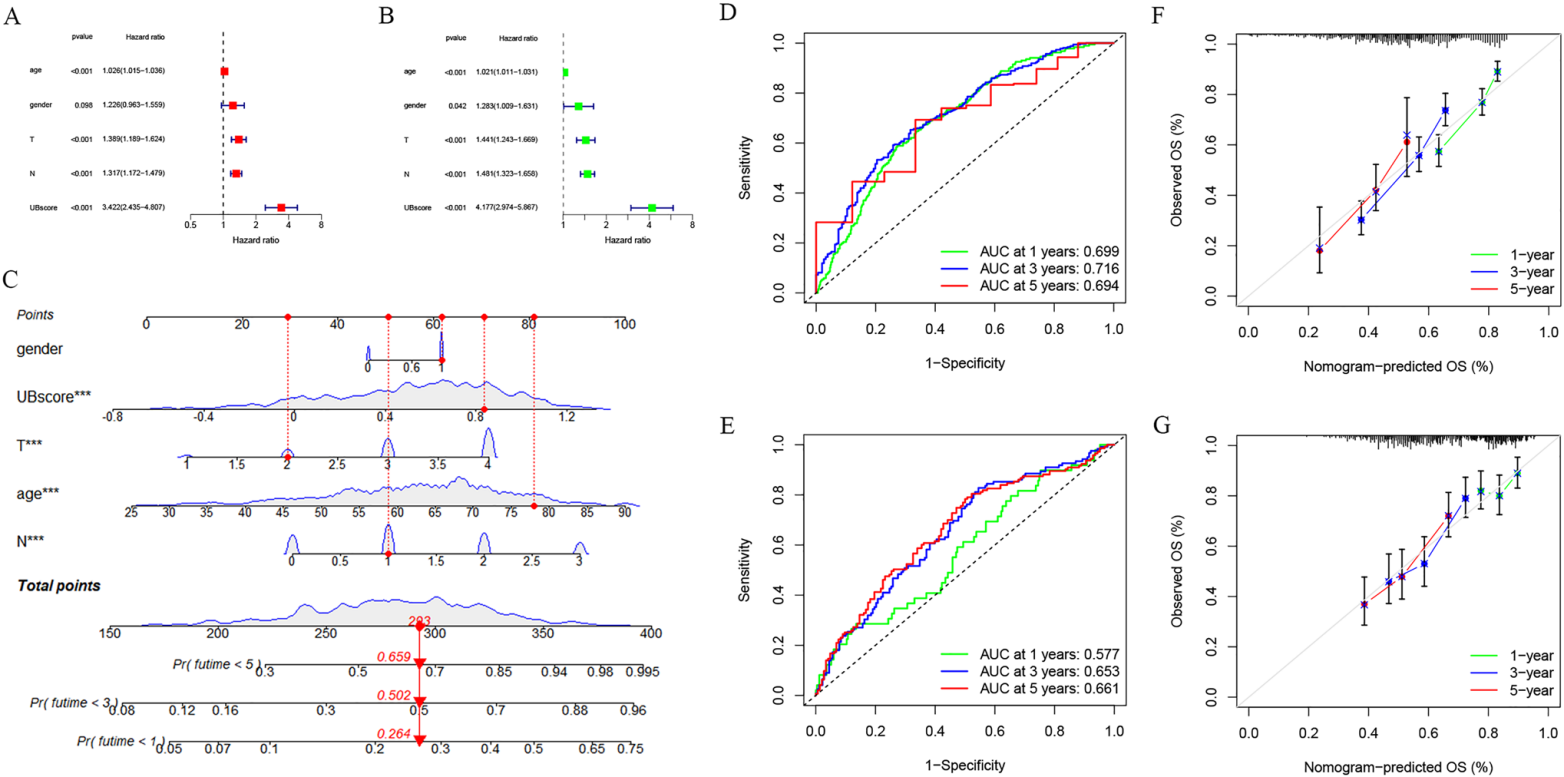
The predictive power of the UBscore. **A** Univariate Cox regression analysis of the UBscore. **B** Multiple Cox regression analysis with the UBscore. **C** Nomogram for predicting the 1-, 3-, and 5-year OS of patients with GC in the TCGA and GSE84437 merged cohorts. **D**, **E** ROC curves for predicting the 1-, 3-, and 5-year ROC curves in the TCGA and GSE84437 merged cohort and the GSE66229 cohort. **F**, **G** Calibration curves of the nomogram for predicting 1-, 3-, and 5-year OS in the TCGA and GSE84437 merged cohort and GSE66229 cohort

To evaluate the predictive value of the prognostic model in clinical practice, we integrated the UBscore with clinical and pathological characteristics, such as age, sex, and TNM stage. Cox regression analyses, both univariate and multivariate, indicated that the UBscore was an independent predictive factor (Fig. 6A, B). To improve forecast accuracy, we developed a nomogram based on the aforementioned prognostic variables (Fig. 6C). The ROC curve demonstrated that the nomograms were effective in predicting the 1-, 3-, and 5-year overall survival of GC patients in the training (TCGA and GSE84437 merged cohort, AUC = 0.699, 0.716, and 0.694, respectively) and validation (GSE66229, AUC = 0.577, 0.653, and 0.661, respectively) cohorts (Fig. 6D, E). The calibration curves for the probability of survival at 1, 3, and 5 years demonstrated the nomogram's accurate predictive abilities (Fig. 6F, G).

### Chemotherapy drug susceptibility analysis

**Fig. 7:**
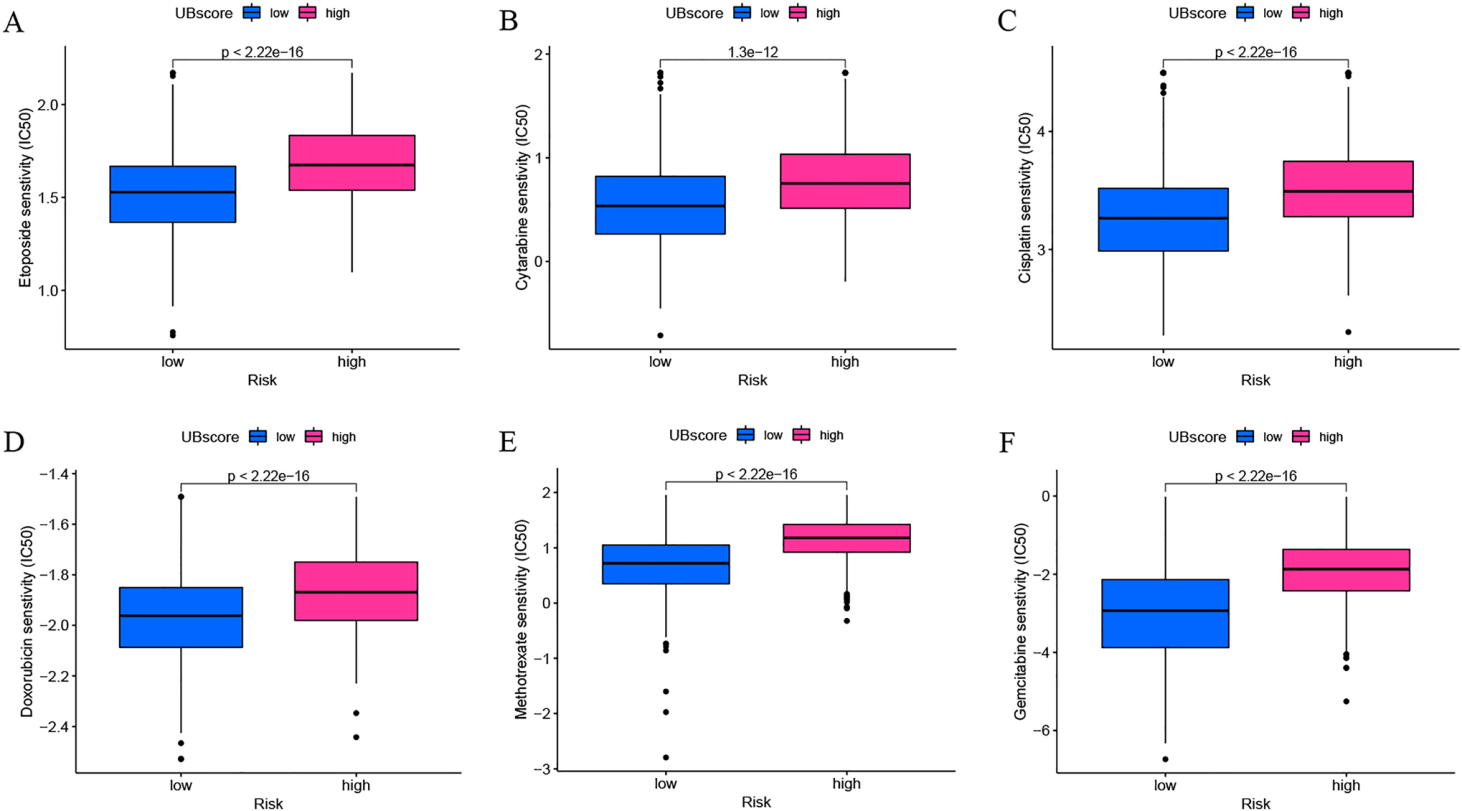
Analysis of the drug susceptibility to chemotherapy in groups with high and low UBscores. **A** Etoposide, **B** ytarabine, **C** cisplatin, **D** doxorubicin, **E** methotrexate, and **F** gemcitabine

We conducted a drug susceptibility analysis to determine the more effective chemotherapy for groups of GC with a high and low UBscore. Patients with a high UBscore exhibited higher IC50 values for etoposide, cytarabine, cisplatin, doxorubicin, methotrexate, and gemcitabine (Fig. 7A–F). These findings confirm the efficacy of the UBscore for predicting medication sensitivity and identifying prospective recipients of certain treatment medicines among GC patients.

### UBscore in immunotherapy prediction in TCGA cohort

**Fig. 8:**
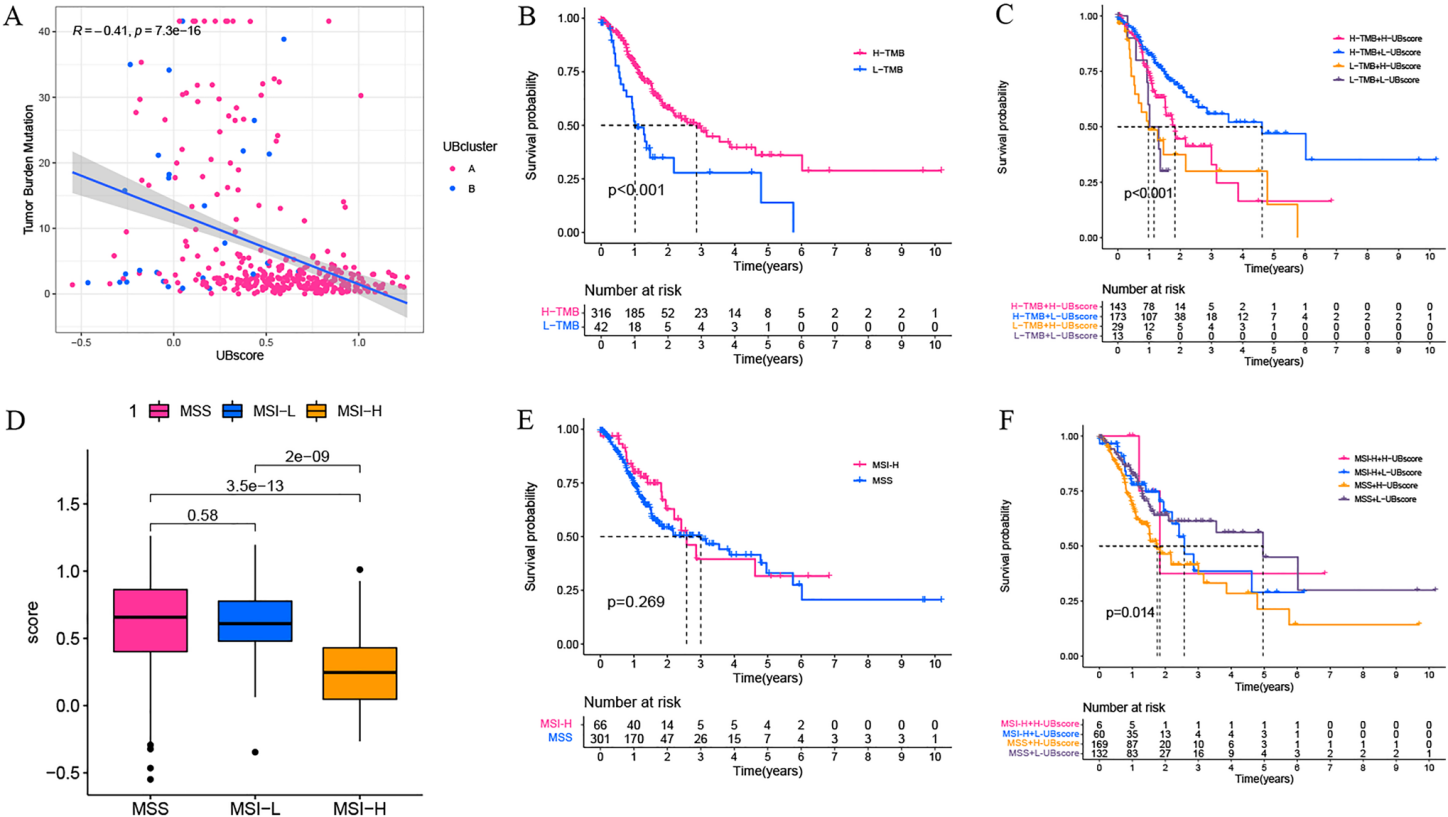
TMB and MSI status of different UBscore groups. **A** TMB was negatively correlated with the UbiquitinScore. **B**, **C** Kaplan–Meier survival analyses were performed on individuals with TMB status or their combination with the UBscore. **D** The UBscore for MSI-H was lower than that of MSS/MSI-L. **E**, **F** K–M analysis based on MSI status or its combination with the UBscore

Experiments were conducted to examine the association between the UBscore and immunotherapeutic indicators such as TMB, MSI, and immune checkpoints. The UBscore had a negative association with TMB (Fig. 8A). K–M analysis based on TMB showed that high-TMB patients had a considerably better OS than low-TMB GC patients (Fig. 8B), particularly when combined with a high UBscore (Fig. 8C). Next, we examined the connection between MSI status and the UBscore. A low UBscore was associated with MSI-H status, whereas a high UBscore was associated with MSS/MSI-L status (Fig. 8D). Although there was no difference in OS between MSI-H and MSS/MSI-L (Fig. 8E), patients with MSS and low UBsore had a significantly better OS than patients in other subgroups (Fig. 8F).

**Fig. 9:**
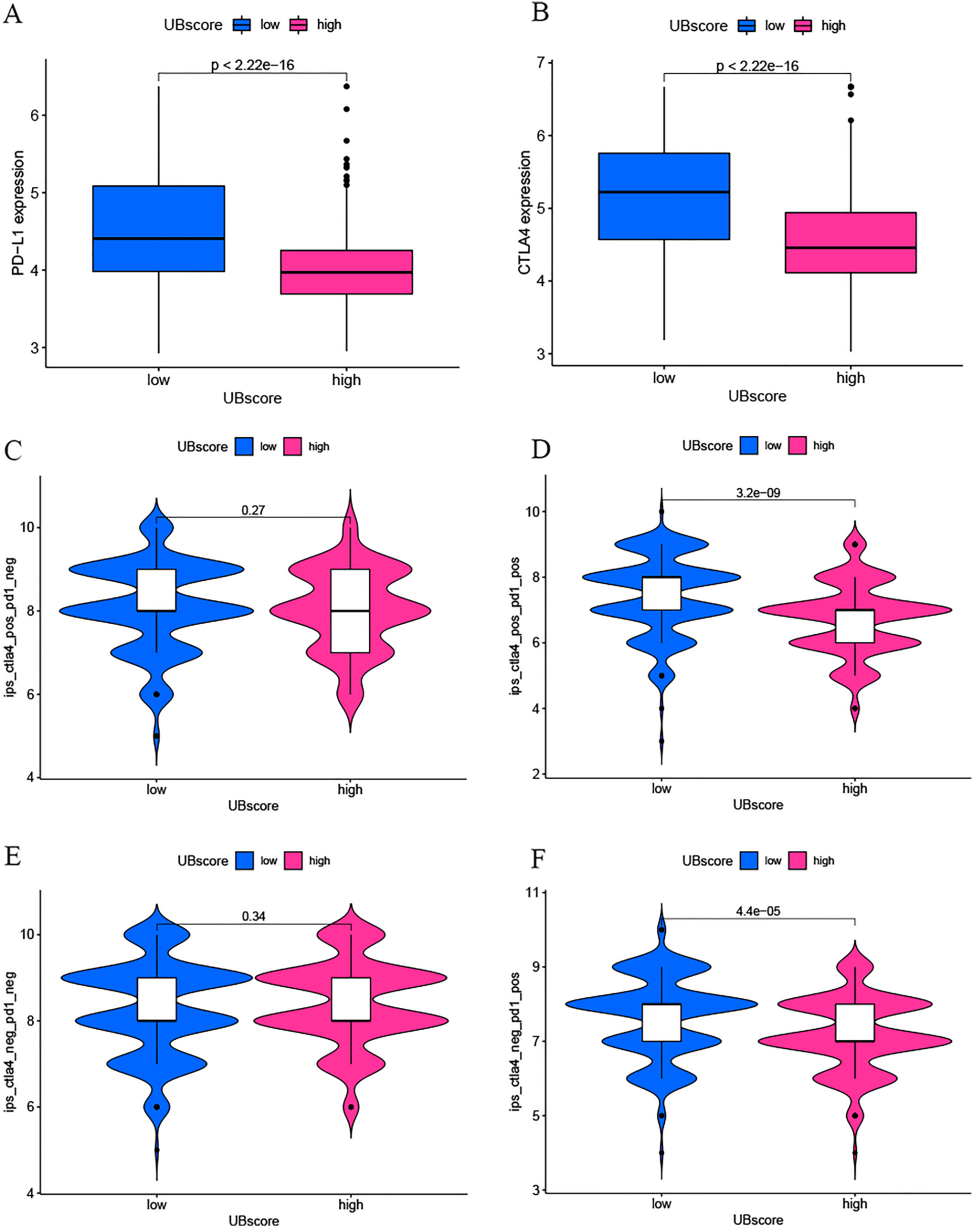
Immunotherapy outcome prediction using the UBscore in the TCGA cohort. **A**, **B** PD-L1 and CTLA4 have higher expression in the low-UBscore group in TCGA. **C**–**F** Different IPS scores between high and low UBscore

IPS scores were downloaded based on the findings of a TCIA database investigation, and their link with the UBscore was also investigated. PD-L1 and CTLA-4 served as immunological checkpoints for the IPS. The expression of PD-L1 and CTLA4 was elevated in the low UBscore group (Fig. 9A, B). When PD-L1 was positive, IPS values were considerably higher in the low UBscore group; however, there was no difference between the low and high UBscore groups when PD-L1 was negative (Fig. 9C–F). In summary, these results suggested that the group with a low UBscore and positive PD-L1 expression was more likely to react favorably to immunotherapy.

### Validation of mass spectrometry and coculturing with NK-92 cells

**Fig. 10:**
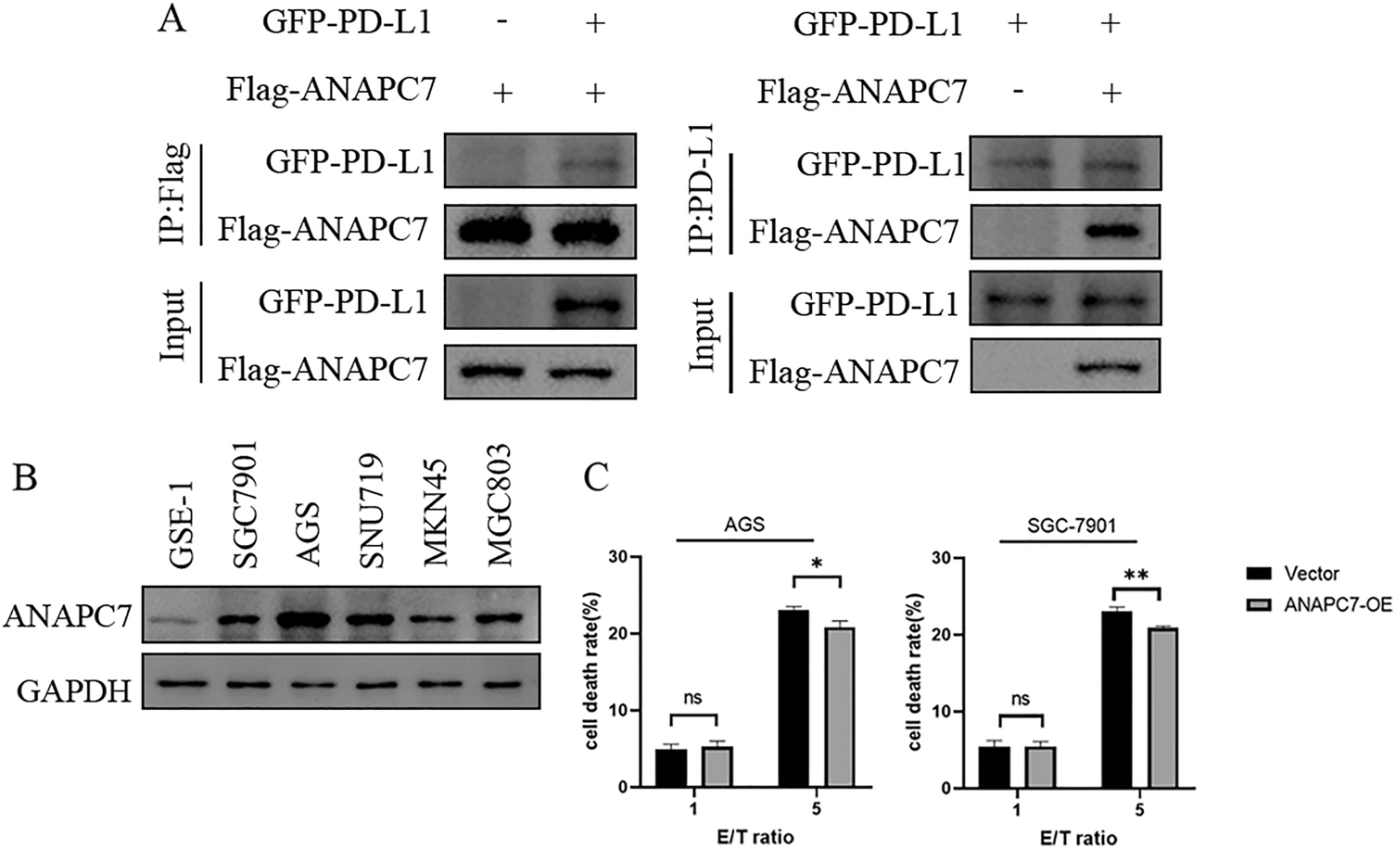
Coimmunoprecipitation and coculture experiment. **A** Analysis of GFP-PD-L1 and Flag-ANAPC7 interactions using CoIP. The indicated plasmids were transiently transfected into HEK293 cells. Flag-ANAPC7 (left) and GFP-PD-L1 (right) were immunoprecipitated and analyzed by western blotting with Flag-tag (left) and PD-L1 antibodies (right). **B** Expression of ANAPC7 in GSE-1 and 5 other gastric cell lines. **C** The release of LDH was used to measure the cytotoxicity of NK-92 cells on AGS and SGC-7901 cells. E/T represents NK-92 cells to cancer cells, *P < 0.05

When GFP-PD-L1 and Flag-ANAPC7 were cotransfected into HEK293 cells, the results depicted in Fig. 10A indicated that they could interact with one another, confirming the mass spectrometry outcome. Analyzing the expression of ANAPC7 in GSE-1 and five GC cell lines revealed that ANAPC7 was significantly upregulated in GC cell lines, particularly AGS (Fig. 10B). To investigate the effect of ANAPC7 on tumor cells, ANAPC7 was overexpressed in AGS cells. NK-92 cells were cocultured with AGS (Vector) and AGS (ANAPC7-OE) cells. LDH release was used to evaluate the cytotoxicity of NK-92 cells against tumor cells. The results demonstrated that overexpression of ANAPC7 diminished the killing ability of NK-92 cells (Fig. 10C).

## Discussion

Immunotherapies, including PD-1/PD-L1 targeted therapy, have recently been shown to be effective cancer treatments. Nonetheless, the clinical response rate with the PD-L1 inhibitor pembrolizumab was just 22% (Brar and Shah 2019). According to some clinical trials, PD-L1 expression status is inadequate in identifying patients who may benefit from PD-1/PD-L1 targeting therapy (Shen and Zhao 2018). Multifactorial biomarkers, such as infiltrating CD8 + T-cell intensity and tumor mutational burden, have been suggested as biomarkers of anti-PD-L1 treatments to solve this dilemma (Topalian et al. 2016).To guide immunotherapy, it is vital to identify novel biomarkers or an effective immune score. Therefore, we utilized PD-L1 protein as bait to identify PD-L1-interacting proteins using LS–MS/MS. The identification of these 123 genes, which were substantially concentrated in the proteasome complex, prompted us to focus on the UPS.

Recent research has shown that ubiquitination and deubiquitination play a significant role in modulating biological activities such as the PD-1/PD-L1 pathway, suggesting that targeting PD-L1 interactors is a potential method for enhancing anticancer immune responses (Burr et al. 2017; Lim et al. 2016). Applying machine learning, we evaluated many UPS genes to identify the crucial genes. Due to their superior capacity to manage big and complicated information, artificial intelligence (AI) systems using machine learning techniques have gained traction in genetic profile evaluation. RF is an algorithm that relies on thousands of decision trees. We identified the 30 most essential UPS genes for diagnosing stomach cancer using random forest. Using neural network-based predictive models, these 30 UPS genes were shown to be highly effective in identifying stomach cancer (AUC = 1).

ANAPC7 was the gene where these 30 UPS genes and 123 PD-L1 interactors intersect. ANAPC7 is a fundamental component of the anaphase-promoting complex (APC), the largest known ubiquitin ligase in human cells (Ferguson et al. 2022). Therefore, we called the subtypes in our study “UBcluster” and the risk score “UBscore”. Although initially recognized for its vital function in mitosis (Craney and Rape 2013), APC also regulates cell differentiation and identity maintenance via various mechanisms (Eguren et al. 2011). As a vital part of APC, ANAPC7 dysregulation contributes to the development of cancers such as acute myeloid leukemia (Rahimi et al. 2015). A study on breast cancer revealed that ANAPC7 expression was elevated, which corresponds to a more malignant tumor condition (Kang et al. 2009). The effect of ANAPC7 on gastric cancer has not been reported. In our investigations, gastric cancer cell lines exhibited increased ANAPC7 expression. Although no change in PD-L1 expression was observed following overexpression of ANAPC7, tumor cells could escape NK-cell killing, indicating that ANAPC7 not only acted as an oncogene to promote carcinogenesis but also encouraged immune escape in tumor cells.

Due to the complexity of tumor biology and its interactions with the immune system, it is doubtful that researching individual components can accurately predict clinical results for immune-targeted therapy (Masucci et al. 2016). Characterizing the tumor microenvironment (TME) based on the quantity, location, and phenotype of tumor-infiltrating lymphocytes and other immune cells gives a more comprehensive knowledge of the many tumor–host interactions. The immune-inflamed phenotype, termed a hot tumor, is characterized by a high level of immune cell infiltration in the TME (Chen and Mellman 2017). Previous studies reported that patients with high immune infiltration had better prognoses for gastric cancer and breast cancer (Wu et al. 2021; Zhang et al. 2020). We divided the patients into two subgroups in the TCGA and GSE84437 merged cohorts using PD-L1 and ANAPC7 as key genes. Consistent with previous research, UBcluster B, with a better prognosis, was the immune-inflamed phenotype, which had a higher proportion of virtually all immune cells, including T cells, B cells, macrophages, and NK cells. In addition, UBcluster B was strongly linked with enrichment pathways associated with immune activation, such as natural killer cell-mediated cytotoxicity and the T-cell receptor signaling pathway.

We built a scoring model (UBscore) to estimate the prognostic risk of patients based on the UBcluster's differentially expressed genes to improve the clinical application. To increase the accuracy of prognostic prediction, we created and verified a nomogram by screening the UBscore, sex, age, and pathological stage. We discovered that the UBscore was an independent prognostic factor based on the results. Our research offered substantial support for the clinical treatment of GC, because the UBscore accounted for the heterogeneity of patients and might also relate ubiquitination to prognosis. Compared to previous models, the PD-L1 and interacting protein-based UBscore might be more useful for immunotherapy prediction.

TMB is an additional measure for assessing patient response to immunotherapy irrespective of PD-L1 expression level (Hodges et al. 2017). According to Panel Sequencing research, patients with increased TMB had longer overall survival, indicating that TMB might be employed as a promising biomarker for GC patients receiving immunotherapy (Kim et al. 2020). In our study, the UBscore had a negative relationship with TMB. Meanwhile, the patients with low UBscore and high TMB had the longest OS, while those with high UBscore and low TMB had the shortest OS. Because of the correlation between the TMB and the UBscore, we can assume that the UBscore also has the potential to predict the effect of immunotherapy.

MSI-H is detected in approximately 22% of patients with gastric cancer (Cancer Genome Atlas Research Network 2014), and MSI-H gastric cancer is associated with a better prognosis than MSI-L/MSS tumors (Jin and Yoon 2016; Zhu et al. 2015). MSI-H is primarily caused by hypermethylation of the MLH1 gene promoter, which results in epigenetic gene silencing. MSI-H status is, in general, a prognostic biomarker for anti-PD-1/PD-L1 medicines in gastric cancer. In our generated UBscore, MSI-H patients had a lower UBscore, and patients with a lower UBscore were predicted to have a higher response to immunotherapy (TCIA), indicating that our UBscore might have good predictive potential for immunotherapy.

According to the current guidelines, chemotherapy remains the predominant drug used to treat gastric cancer. We wanted to determine whether gastric cancer patients could benefit from both immunotherapy and chemotherapy. Our study showed that patients with low UBscore were more sensitive to chemotherapy drugs, such as etoposide, cytarabine, cisplatin, doxorubicin, methotrexate, and gemcitabine. These results imply that patients with low UBscore might achieve optimal treatment outcomes when receiving both chemotherapy and immunotherapy.

## Conclusion

Overall, we found a new protein binding to PDL1 by mass spectrometry and machine learning that helped to distinguish immune infiltration in GC patients. Meanwhile, the constructed UBscore had certain prognostic predictive values, which provided guidance for immunotherapy and chemotherapy.

## Supplementary Information

Below is the link to the electronic supplementary material.

Supplementary file1 (XLSX 13 KB)

## Data Availability

The datasets generated during and/or analyzed during the current study are available from the corresponding author on reasonable request.

## References

[CR1] Brar G, Shah MA (2019). The role of pembrolizumab in the treatment of PD-L1 expressing gastric and gastroesophageal junction adenocarcinoma. Therap Adv Gastroenterol.

[CR2] Burr ML, Sparbier CE, Chan YC, Williamson JC, Woods K, Beavis PA (2017). CMTM6 maintains the expression of PD-L1 and regulates anti-tumour immunity. Nature.

[CR3] Cancer Genome Atlas Research Network (2014). Comprehensive molecular characterization of gastric adenocarcinoma. Nature.

[CR4] Chen DS, Mellman I (2017). Elements of cancer immunity and the cancer-immune set point. Nature.

[CR5] Craney A, Rape M (2013). Dynamic regulation of ubiquitin-dependent cell cycle control. Curr Opin Cell Biol.

[CR6] Eguren M, Manchado E, Malumbres M (2011). Non-mitotic functions of the anaphase-promoting complex. Semin Cell Dev Biol.

[CR7] Favaro G, Romanello V, Varanita T, Andrea DM, Morbidoni V, Tezze C (2019). DRP1-mediated mitochondrial shape controls calcium homeostasis and muscle mass. Nat Commun.

[CR8] Ferguson CJ, Urso O, Bodrug T, Gassaway BM, Watson ER, Prabu JR (2022). APC7 mediates ubiquitin signaling in constitutive heterochromatin in the developing mammalian brain. Mol Cell.

[CR9] Fuchs CS, Doi T, Jang RW, Muro K, Satoh T, Machado M (2018). Safety and efficacy of pembrolizumab monotherapy in patients with previously treated advanced gastric and gastroesophageal junction cancer: phase 2 Clinical KEYNOTE-059 trial. JAMA Oncol.

[CR10] Giatromanolaki A, Koukourakis IM, Balaska K, Mitrakas AG, Harris AL, Koukourakis MI (2019). Programmed death-1 receptor (PD-1) and PD-ligand-1 (PD-L1) expression in non-small cell lung cancer and the immune-suppressive effect of anaerobic glycolysis. Med Oncol.

[CR11] Hiscott J, Grandvaux N, Sharma S, Tenoever BR, Servant MJ, Lin R (2003). Convergence of the NF-kappaB and interferon signaling pathways in the regulation of antiviral defense and apoptosis. Ann N Y Acad Sci.

[CR12] Hodges TR, Ott M, Xiu J, Gatalica Z, Swensen J, Zhou S (2017). Mutational burden, immune checkpoint expression, and mismatch repair in glioma: implications for immune checkpoint immunotherapy. Neuro Oncol.

[CR13] Hooijman PE, Beishuizen A, Witt CC, de Waard MC, Girbes AR, Spoelstra-de MA (2015). Diaphragm muscle fiber weakness and ubiquitin-proteasome activation in critically ill patients. Am J Respir Crit Care Med.

[CR14] Hu X, Wang J, Chu M, Liu Y, Wang ZW, Zhu X (2021). Emerging Role of Ubiquitination in the Regulation of PD-1/PD-L1 in Cancer Immunotherapy. Mol Ther.

[CR15] Jin Z, Yoon HH (2016). The promise of PD-1 inhibitors in gastro-esophageal cancers: microsatellite instability vs. PD-L1. J Gastrointest Oncol.

[CR16] Joshi SS, Badgwell BD (2021). Current treatment and recent progress in gastric cancer. CA Cancer J Clin.

[CR17] Kang Y, Kim JH, Lee TH, Kim TS, Jung WH, Chung HC (2009). Expression of anaphase-promoting complex7 in fibroadenomas and phyllodes tumors of breast. Hum Pathol.

[CR18] Kim J, Kim B, Kang SY, Heo YJ, Park SH, Kim ST (2020). Tumor mutational burden determined by panel sequencing predicts survival after immunotherapy in patients with advanced gastric cancer. Front Oncol.

[CR19] Liao X, Li G, Cai R, Chen R (2022). A review of emerging biomarkers for immune checkpoint inhibitors in tumors of the gastrointestinal tract. Med Sci Monit.

[CR20] Lim SO, Li CW, Xia W, Cha JH, Chan LC, Wu Y (2016). Deubiquitination and stabilization of PD-L1 by CSN5. Cancer Cell.

[CR21] Masucci GV, Cesano A, Hawtin R, Janetzki S, Zhang J, Kirsch I (2016). Validation of biomarkers to predict response to immunotherapy in cancer: volume I - pre-analytical and analytical validation. J Immunother Cancer.

[CR22] Mezzadra R, Sun C, Jae LT, Gomez-Eerland R, de Vries E, Wu W (2017). Identification of CMTM6 and CMTM4 as PD-L1 protein regulators. Nature.

[CR23] Ndoja A, Reja R, Lee SH, Webster JD, Ngu H, Rose CM (2020). Ubiquitin ligase COP1 suppresses neuroinflammation by degrading c/EBPbeta in microglia. Cell.

[CR24] Osterloh P, Linkemann K, Tenzer S, Rammensee HG, Radsak MP, Busch DH (2006). Proteasomes shape the repertoire of T cells participating in antigen-specific immune responses. Proc Natl Acad Sci U S A.

[CR25] Owens T, Benmamar-Badel A, Wlodarczyk A, Marczynska J, Morch MT, Dubik M (2020). Protective roles for myeloid cells in neuroinflammation. Scand J Immunol.

[CR26] Pohl C, Dikic I (2019). Cellular quality control by the ubiquitin-proteasome system and autophagy. Science.

[CR27] Puvar K, Luo ZQ, Das C (2019). Uncovering the structural basis of a new twist in protein ubiquitination. Trends Biochem Sci.

[CR28] Rahimi H, Ahmadzadeh A, Yousef-amoli S, Kokabee L, Shokrgozar MA, Mahdian R (2015). The expression pattern of APC2 and APC7 in various cancer cell lines and AML patients. Adv Med Sci.

[CR29] Sanmamed MF, Chen L (2018). A paradigm shift in cancer immunotherapy: from enhancement to normalization. Cell.

[CR30] Sharma A, Khan H, Singh TG, Grewal AK, Najda A, Kawecka-Radomska M (2021). Pharmacological modulation of ubiquitin-proteasome pathways in oncogenic signaling. Int J Mol Sci.

[CR31] Shen X, Zhao B (2018). Efficacy of PD-1 or PD-L1 inhibitors and PD-L1 expression status in cancer: meta-analysis. BMJ.

[CR32] Sung H, Ferlay J, Siegel RL, Laversanne M, Soerjomataram I, Jemal A (2021). Global cancer statistics 2020: GLOBOCAN estimates of incidence and mortality worldwide for 36 cancers in 185 countries. CA Cancer J Clin.

[CR33] Topalian SL, Taube JM, Anders RA, Pardoll DM (2016). Mechanism-driven biomarkers to guide immune checkpoint blockade in cancer therapy. Nat Rev Cancer.

[CR34] Wu X, Liu G, Liu R, He J, Wang G, Zhang H (2020). Expression of ubiquitin-conjugating enzyme E2T in colorectal cancers and clinical implications. Oncol Lett.

[CR35] Wu J, Zhu Y, Luo M, Li L (2021). Comprehensive analysis of pyroptosis-related genes and tumor microenvironment infiltration characterization in breast cancer. Front Immunol.

[CR36] Yang Y, Liu W, Hu D, Su R, Ji M, Huang Y (2020). HIV-1 nef interacts with LMP7 to attenuate immunoproteasome formation and major histocompatibility complex class I antigen presentation. Mbio.

[CR37] Zhang B, Wu Q, Li B, Wang D, Wang L, Zhou YL (2020). m(6)A regulator-mediated methylation modification patterns and tumor microenvironment infiltration characterization in gastric cancer. Mol Cancer.

[CR38] Zhu L, Li Z, Wang Y, Zhang C, Liu Y, Qu X (2015). Microsatellite instability and survival in gastric cancer: a systematic review and meta-analysis. Mol Clin Oncol.

[CR39] Zhu B, Zhu L, Xia L, Xiong Y, Yin Q, Rui K (2020). Roles of ubiquitination and deubiquitination in regulating dendritic cell maturation and function. Front Immunol.

